# Body mass index and body composition in relation to 14 cardiovascular conditions in UK Biobank: a Mendelian randomization study

**DOI:** 10.1093/eurheartj/ehz388

**Published:** 2019-06-13

**Authors:** Susanna C Larsson, Magnus Bäck, Jessica M B Rees, Amy M Mason, Stephen Burgess

**Affiliations:** 1 Unit of Cardiovascular and Nutritional Epidemiology, Institute of Environmental Medicine, Karolinska Institutet, Nobels väg 13, SE-17177 Stockholm, Sweden; 2 Department of Surgical Sciences, Uppsala University, Uppsala, Sweden; 3 Department of Medicine, Center for Molecular Medicine, Karolinska Institutet, Stockholm, Sweden; 4 Heart and Vascular Theme – Division of Valvular and Coronary Disease, Karolinska University Hospital, Stockholm, Sweden; 5 Department of Public Health and Primary Care, University of Cambridge, Cambridge, UK; 6 Edinburgh Clinical Trials Unit, Usher, Institute of Population Health Sciences and Informatics, University of Edinburgh, Edinburgh, UK; 7 MRC Biostatistics Unit, University of Cambridge, UK

**Keywords:** Adiposity, Body composition, Body mass index, Cardiovascular disease, Mendelian randomization, Obesity

## Abstract

**Aims:**

The causal role of adiposity for several cardiovascular diseases (CVDs) is unclear. Our primary aim was to apply the Mendelian randomization design to investigate the associations of body mass index (BMI) with 13 CVDs and arterial hypertension. We also assessed the roles of fat mass and fat-free mass on the same outcomes.

**Methods and results:**

Single-nucleotide polymorphisms associated with BMI and fat mass and fat-free mass indices were used as instrumental variables to estimate the associations with the cardiovascular conditions among 367 703 UK Biobank participants. After correcting for multiple testing, genetically predicted BMI was significantly positively associated with eight outcomes, including and with decreasing magnitude of association: aortic valve stenosis, heart failure, deep vein thrombosis, arterial hypertension, peripheral artery disease, coronary artery disease, atrial fibrillation, and pulmonary embolism. The odds ratio (OR) per 1 kg/m^2^ increase in BMI ranged from 1.06 [95% confidence interval (CI) 1.02–1.11; *P *=* *2.6 × 10^−3^] for pulmonary embolism to 1.13 (95% CI 1.05–1.21; *P *=* *1.2 × 10^−3^) for aortic valve stenosis. There was suggestive evidence of positive associations of genetically predicted fat mass index with nine outcomes (*P *<* *0.05). The strongest magnitude of association was with aortic valve stenosis (OR per 1 kg/m^2^ increase in fat mass index 1.46, 95% CI 1.13–1.88; *P *=* *3.9 × 10^−3^). There was suggestive evidence of inverse associations of fat-free mass index with atrial fibrillation, ischaemic stroke, and abdominal aortic aneurysm.

**Conclusion:**

This study provides evidence that higher BMI and particularly fat mass index are associated with increased risk of aortic valve stenosis and most other cardiovascular conditions.

**Figure:**
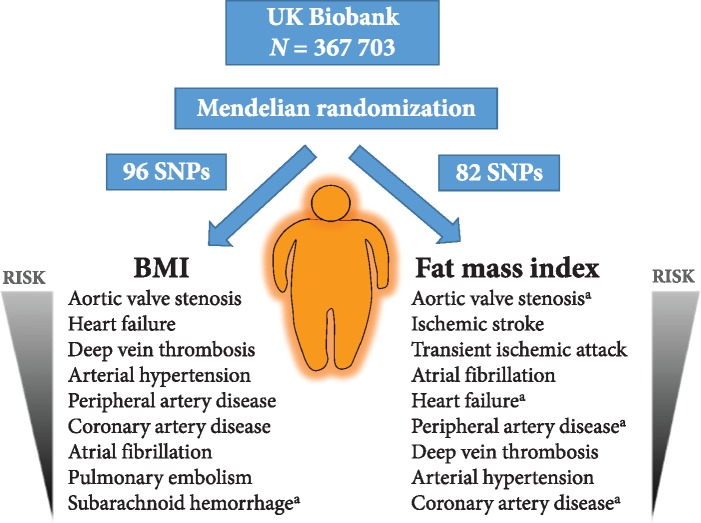

**Translational perspective**

Mendelian randomization (MR) is an epidemiologic method that exploits genetic variants as proxy indicators for the modifiable risk factor to determine whether the risk factor is causally associated with the disease. This study design is less susceptible to confounding and reverse causality compared to conventional observational studies. In this MR study, genetically predicted body mass index was significantly positively associated with 8 of the 14 assessed cardiovascular conditions, including aortic valve stenosis, heart failure, deep vein thrombosis, arterial hypertension, peripheral artery disease, coronary artery disease, atrial fibrillation, and pulmonary embolism. Genetically predicted fat mass index was also positively associated with most cardiovascular diseases, with the strongest magnitude of association with aortic valve stenosis. These results support current guidelines that overweight and obese individuals should aim for a reduction in body weight and fat mass in order to reduce cardiovascular disease risk.

## Introduction

The prevalence and disease burden of high body mass index (BMI) are increasing globally.^1^ High BMI along with smoking, poor diet, high blood pressure, alcohol, and drug use are the leading causes of years of life lost.^2^ Over two-thirds of deaths attributable to high BMI are due cardiovascular disease (CVD).^1^ While a number of studies have indicated that BMI may be a causal risk factor for coronary heart disease,^3–6^ uncertainties remains about the causal status of BMI for several other CVDs. In particular, there is a scarcity of data on the causal role of BMI for aortic valve stenosis, aortic aneurysms, deep vein thrombosis, pulmonary embolism, and intracerebral and subarachnoid haemorrhages. Available evidence on BMI in relation to those CVDs originates from observational studies,^7–11^ which are susceptible to confounding and reverse causation bias and thus cannot infer causality.

Given the uncertainties about the causal role of BMI for several CVDs, we applied the Mendelian randomization (MR) design to evaluate and compare the associations of BMI, as a measurement of overall adiposity, with 13 CVDs and arterial hypertension. In complementary analyses, we investigated the roles of fat mass and fat-free mass indices for the same conditions.

## Methods

### Study design

Mendelian randomization is an epidemiologic method based on instrumental variable analysis and utilizes genetic variants, usually single-nucleotide polymorphisms (SNPs), associated with the modifiable risk factor (e.g. BMI) as proxy indicators to infer causality.^12^ The MR design reduces bias due to confounding as alleles are randomly assorted at conception and prevents reverse causation bias because disease cannot affect genotype.

### Data sources and single-nucleotide polymorphism selection

Genetic association estimates with the outcomes were obtained from the UK Biobank.^13^ During 2006 and 2010, about 500 000 community-dwelling adults, aged 40–69 years, across the UK were enrolled into the cohort (https://www.ukbiobank.ac.uk/).^13^ To reduce confounding by ancestry, we restricted the analytic cohort to individuals of White-British descent. After exclusion of related individuals (third-degree relatives or closer), low call rate (3 or more standard deviations from the mean), and excess heterozygosity, 367 703 individuals remained for analysis. We used follow-up data until 17 February 2016, and defined the outcomes based on electronic health, hospital procedure codes, and self-reported information confirmed by interview with a nurse (Supplementary material online, *Table S1*). To be able to determine differential cardiovascular risk associated with adiposity, we analysed a broad range of CVDs, including cerebrovascular diseases (ischaemic stroke, transient ischaemic attack, intracerebral haemorrhage, and subarachnoid haemorrhage), aneurysms (abdominal and thoracic aortic aneurysm), thrombo-embolic diseases (deep vein thrombosis and pulmonary embolism), and other CVDs (coronary artery disease, aortic valve stenosis, atrial fibrillation, heart failure, and peripheral vascular disease) as well as arterial hypertension. Logistic regression, adjusted for 10 genetic principal components, was applied to estimate the genetic associations with the outcomes. The North West Multicenter Research Ethics Committee approved the UK Biobank study. All participants provided written informed consent to participate in the study.

As instrumental variables we used SNPs associated with BMI at the genome-wide significance threshold (*P *<* *5 × 10^−8^) in a meta-analysis of genome-wide association studies, including 339 224 individuals.^14^ One of the BMI-associated SNPs was unavailable in UK Biobank, leaving 96 SNPs as instrumental variables (Supplementary material online, *Table S2*). For fat mass and fat-free mass (assessed using bioelectrical impedance technique), we considered the 98 SNPs associated with body composition at genome-wide significance among 362 499 UK Biobank participants.^15^ Of those SNPs, we included 82 that had an imputation quality score greater than 0.8 and were in Hardy–Weinberg equilibrium (Supplementary material online, *Table S3*). We calculated fat mass index analogously to BMI as fat mass divided by height squared, and fat-free mass similarly. Genetic associations with BMI were taken from the published meta-analysis^14^ and do not include UK Biobank participants, whereas genetic associations with fat mass and fat-free mass indices were estimated in UK Biobank by linear regression with adjustment for 10 genetic principal components.

### Statistical analyses

The principal analyses for BMI were conducted using inverse-variance weighted meta-analysis (under a random-effects model), combining the instrumental variable ratio estimates (i.e. the beta coefficients for the SNP–outcome association divided by the beta-coefficient for the SNP–BMI association) across the 96 BMI-associated SNPs. As a first sensitivity analysis, we excluded potential outlier SNPs (*P *<* *0.10) identified using the MR Pleiotropy RESidual Sum and Outlier (MR-PRESSO) method.^16^ In a second sensitivity analysis, we applied the weighted median approach, which can provide valid estimates if at least 50% of the information in the analysis comes from SNPs that are valid instrumental variables.^17^ A fundamental assumption of an MR analysis is that the instrumental variables must only be associated with the outcome through the risk factor under study and not via any other causal pathway, so called pleiotropy. In a third sensitivity analysis, we used the MR-Egger method to assess directional pleiotropy.^18^ Independent associations of genetically predicted fat mass and fat-free mass indices with the outcomes were analysed using multivariable MR, with the two indices included in the same regression model. All reported odds ratios (OR) are expressed per 1 kg/m^2^ increment of each body composition measure. All statistical tests are two-tailed. To account for multiple testing in our primary analyses of BMI in relation to the 14 outcomes, we used a Bonferroni-corrected threshold of *P *<* *3.6 × 10^−3^ (*α* = 0.05/14 outcomes). Associations with *P*-values between 3.6 × 10^−3^ and 0.05 were considered suggestive evidence of associations, requiring confirmation. The analyses were carried out using Stata (StataCorp, College Station, TX, USA) and R (R Foundation). Statistical power was estimated with the method proposed by Brion *et al.*^19^ and is shown in the Supplementary material online, *Table S4*.

## Results

### Participants

The mean age of the 367 703 participants included in the present analysis was 57.2 years (5th to 95th percentile: 42.9 to 68.6 years) and 46% were men. The proportion of current smokers and alcohol drinkers were respectively 10.3% and 93.4%. The 96 BMI-related SNPs explained 1.6% of the variance in BMI (corresponding to an F statistic of 61), whereas the 82 SNPs for body composition explained 0.8% of the variance in fat mass index (F statistic 52) and 0.7% of the variance in fat-free mass index (F statistic 45). Conditional F statistics^20^ were 30 for fat mass index and 26 for fat-free mass index.

### Body mass index

**Figure 1:**
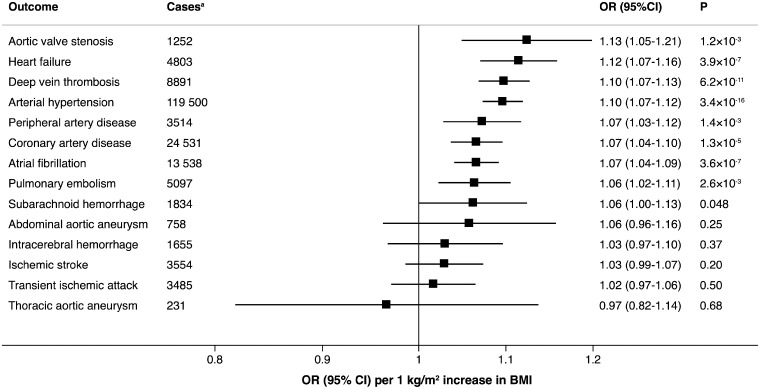
Associations of genetically predicted 1 kg/m^2^ increase in body mass index with 14 cardiovascular conditions in UK Biobank. ^a^Total number of individuals is 367 703.

Genetically predicted BMI was significantly positively associated with 8 of the 14 outcomes, including and with decreasing magnitude of association: aortic valve stenosis, heart failure, deep vein thrombosis, arterial hypertension, peripheral artery disease, coronary artery disease, atrial fibrillation, and pulmonary embolism (*Figure 1*). The OR per genetically predicted 1 kg/m^2^ increase of BMI ranged from 1.06 [95% confidence interval (CI) 1.02–1.11; *P *=* *2.6 × 10^−3^] for pulmonary embolism to 1.13 (95% CI 1.05–1.21; *P *=* *1.2 × 10^−3^) for aortic valve stenosis (*Figure 1*). There was suggestive evidence of a positive association between genetically predicted BMI and subarachnoid haemorrhage, whereas no association was observed between BMI and abdominal or thoracic aortic aneurysm, ischaemic stroke, transient ischaemic attack, or intracerebral haemorrhage (*Figure 1*).

The MR-PRESSO method identified one outlier SNP for heart failure, six outlier SNPs for coronary artery disease, and 11 outlier SNPs for arterial hypertension. Outlier-correction did not materially change the OR estimates for heart failure (1.13; 95% CI 1.08–1.17), coronary artery disease (1.08; 95% CI 1.06–1.10), or arterial hypertension (1.10; 95% CI 1.08–1.12). No outlier SNPs were identified in the MR-PRESSO analysis of the other outcomes.

Similar OR estimates as in the standard MR analysis (inverse-variance weighted method) but with lower precision were obtained from the weighted median analysis (Supplementary material online, *Table S5*). For most outcomes, the MR-Egger analysis revealed consistent estimates but of low precision, and without indication of directional pleiotropy (Supplementary material online, *Table S5*). The exceptions were coronary artery disease, atrial fibrillation, and transient ischaemic attack where directional pleiotropy was detected (Supplementary material online, *Table S5*).

### Fat mass and fat-free mass indices

**Figure 2:**
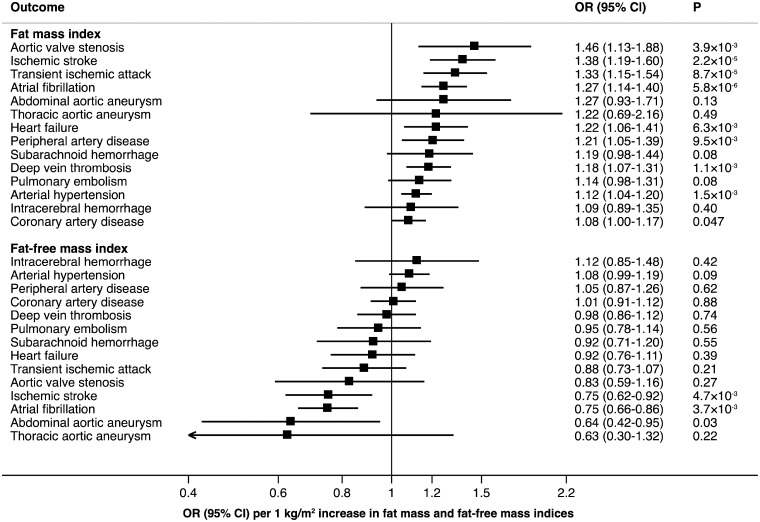
Associations of genetically predicted 1 kg/m^2^ increase in fat mass and fat-free mass indices with 14 cardiovascular conditions in UK Biobank.

In our complementary analyses of fat mass and fat-free mass indices, we found significant or suggestive evidence of inverse associations of genetically predicted fat mass index with 9 of the 14 outcomes (*Figure 2*; Take home figure). Similar to BMI, the strongest magnitude of association was observed between fat mass index and aortic valve stenosis (OR 1.46, 95% CI 1.13–1.88; *P *=* *3.9 × 10^−3^). There was significant or suggestive evidence that genetically predicted fat-free mass index was inversely associated with atrial fibrillation, ischaemic stroke, and abdominal aortic aneurysm (*Figure 2*).

**Take home figure:**
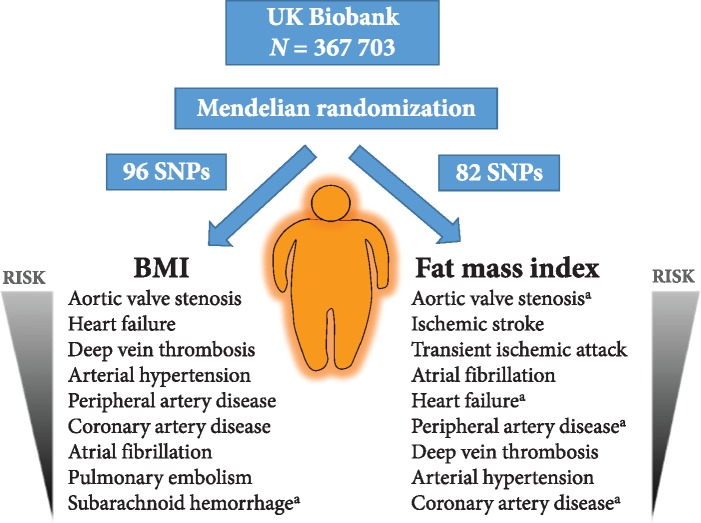
Observed associations of BMI and fat mass index with cardiovascular conditions in UK Biobank. ^a^Significant association at the *P *<* *0.05 level; all other associations are significant at a Bonferroni threshold of *P *<* *3.6 × 10^−3^ (corrected for 14 outcomes).

## Discussion

### Principal findings

In this MR study, we assessed the causal roles of BMI and body composition for a wide range of cardiovascular conditions and found evidence that higher BMI and especially fat mass index are associated with increased risk of most outcomes (*Take home figure*). A novel finding is a strong and consistent positive association between genetically predicted BMI and aortic valve stenosis. In contrast, there was significant or suggestive evidence of inverse associations of fat-free mass index with atrial fibrillation, ischaemic stroke, and abdominal aortic aneurysm.

### Comparisons with other studies

A few conventional observational studies have investigated the association of self-reported or measured BMI with incidence of aortic valve stenosis. Using data from two population-based cohort studies comprising over 70 000 Swedish adults, we previously showed a dose–response positive relationship between self-reported BMI and risk of aortic valve stenosis.^9^ A positive association between BMI and risk of aortic valve stenosis has also been observed in smaller cohort studies of 3243 to 5079 individuals.^7^^,^^8^ To the best of our knowledge, the present study is the first to assess and corroborate BMI as a causal risk factor for aortic valve stenosis using the MR design. The association appeared to be driven by fat mass index, whereas no association was observed with fat-free mass index.

We are not aware of any previous MR study assessing the association between BMI and abdominal aortic aneurysm. Our null results for genetically predicted BMI and abdominal aortic aneurysm are consistent with findings from a cohort study of 63 655 Swedish adults showing no association between baseline BMI and incidence of abdominal aortic aneurysm.^11^ Likewise, a meta-analysis of observational studies showed no difference in BMI in patients with abdominal aortic aneurysm compared with individuals without the disease.^21^ Studies on BMI in relation to thoracic aortic aneurysm are lacking or scarce, and results from our study based on few cases suggest no association.

This MR study confirms and extends the results of previous smaller MR studies showing that genetically higher BMI is associated with increased risk of heart failure in the ENGAGE consortium (*n* = 19 384 individuals),^3^ venous thromboembolism (deep vein thrombosis and pulmonary embolism combined) in the INVENT consortium (*n* = 52 632 individuals),^22^ and peripheral artery disease in a study of 11 477 Chinese adults.^23^ Furthermore, results of our primary analyses showing positive associations of genetically predicted BMI with coronary artery disease and atrial fibrillation among UK Biobank participants are consistent with results of previous MR studies based on data from other populations^3–5^^,^^24^ and 119 859 participants from the UK Biobank.^6^

As in previous MR analyses based on data from the METASTROKE consortium,^4^^,^^25^ we found no significant association between BMI and ischaemic stroke. Nevertheless, we observed a positive association of fat mass index and an inverse association with fat-free mass index with ischaemic stroke. We also found a suggestive positive association of genetically predicted BMI with subarachnoid haemorrhage but not intracerebral haemorrhage. The MR results for BMI and stroke types diverge from observational data.^10^ Results of a large cohort study of British women and a meta-analysis of observational studies showed that BMI is significantly positively associated with risk of ischaemic stroke in individuals of both European and Asian ancestries (relative risks of 1.21–1.35 per 5 kg/m^2^ increase of BMI).^10^ Body mass index was shown to be inversely associated with risk of subarachnoid haemorrhage, intracerebral haemorrhage, and haemorrhagic stroke as a whole in European-descent individuals (relative risks of 0.89–0.91 per 5 kg/m^2^ increase of BMI) but positively associated with any haemorrhagic stroke in Asians (relative risk of 1.16 per 5 kg/m^2^ increase of BMI).^10^

With regard to body composition, our results for genetically predicted fat mass index and atrial fibrillation confirm the results of a recent MR study showing that fat mass was positively associated with atrial fibrillation.^26^ We are not aware of any other MR study of fat mass and fat-free mass indices in relation to other cardiovascular conditions.

### Potential mechanisms

Genetically predicted BMI is associated with higher left ventricular hypertrophy and increased concentrations of triglycerides, glucose, insulin, and interleukin 6 (an inflammatory marker),^6^ which may contribute to the observed associations with CVD. Arterial hypertension is another possible mediator of the association between BMI and CVD. Genetically predicted BMI was shown to be strongly associated with arterial hypertension as well as systolic and diastolic blood pressure in a previous MR study.^4^ A strong positive association between BMI and arterial hypertension was also observed in the present MR study. Findings from our MR study further suggest that the association between BMI and CVD is related to fat mass index and not fat-free mass index, which might even be associated with lower risk of some CVDs.

### Strengths and limitations

A chief strength of this study is that we could assess and compare associations of adiposity measures with a wide range of CVDs in the same study population using the MR study design. Because alleles are randomly assorted and fixed at conception, biases due to confounding and reverse causality are mitigated in an MR analysis. Hence, the obesity paradox, which implies that obesity is protective and associated with better survival in elderly individuals or those with chronic disease, unlikely affected the results in this study. A further strength is that we could confine the study population to individuals of European descent. This restriction reduces bias due to population stratification.

A potential limitation in any MR analysis is pleiotropy, that is, where a genetic variant associate with more than one phenotype. In this MR study, evidence of directional pleiotropy was found in the analyses of BMI in relation to coronary artery disease, atrial fibrillation, and transient ischaemic attack but not in the analyses of the other outcomes. A possible explanation for the observed pleiotropic effects is that some of the BMI-associated SNPs may be more strongly associated with fat mass whether other SNPs are more strongly associated with fat-free mass.

Another shortcoming is that the number of cases was few for some outcomes. We thus cannot rule out that we may have overlooked weak associations due to insufficient power. A further limitation of the analysis is the potential for weak instrument bias in the analyses for fat mass and fat-free mass indices. However, F statistics for these exposures were well over 10, meaning that the magnitude of bias is likely to be small.

Finally, the lack of information on aortic stenosis severity should be acknowledged as a possible limitation. However, previous results showed similar associations for obesity with total aortic valve stenosis and aortic valve stenosis requiring aortic valve replacement,^9^ indicating an applicability of the results over different degrees of aortic stenosis severity.

### Genetics and lifestyle

While genome-wide association and twin studies support a role of genes in the determination of BMI,^14^^,^^27^ lifestyle factors, including diet and physical activity, are the major determinants of BMI. A healthy diet is recommended as a cornerstone of CVD prevention, and energy intake should be limited to the amount of energy required to maintain or obtain a healthy weight (i.e. BMI between 20 and 25 kg/m^2^).^28^

### Future directions

The exploration of a number of different CVD outcomes in this study provides arguments for a differential CVD risk associated with adiposity, which may be of particular importance in terms of risk stratification in different CVD patient groups. For example, since aortic valve stenosis share some, but not all, risk factors with coronary artery disease, differential preventive measures should potentially be considered. The present study adds important information to this field of research by identifying causal adiposity-associated risk for aortic valve stenosis and provide a first suggestion that weight loss/control should be evaluated for slowing down aortic stenosis progression. Other implications of the present study findings include for example that further large studies are necessary to determine whether adiposity is a causal risk factor for different stroke types. There is also a need for further research to better understand the mechanisms underpinning the association between BMI and each CVD outcome.

## Conclusions

This comprehensive MR analysis of about 370 000 individuals of European ancestry provides evidence that higher BMI and particularly fat mass index are associated with increased risk of aortic valve stenosis and most other cardiovascular conditions. These findings support current guidelines that overweight and obese individuals should aim for a reduction in body weight in order to reduce CVD risk.^28^

## Supplementary Material

ehz388_Supplementary_Data

## Notes

**See page 227 for the editorial comment on this article (doi: 10.1093/eurheartj/ehz569)**
