# Supplementary material for: Body mass index and body composition in relation to 14 cardiovascular conditions in UK Biobank: a Mendelian randomization study
Source: Eur Heart J. 2019 Jun 13;41(2):221–6. doi: 10.1093/eurheartj/ehz388 (PMC6945523; doi:10.1093/eurheartj/ehz388)
Supplement: ehz388_Supplementary_Data [file ehz388_supplementary_data.docx]

**Supplementary material online:**

Body mass index and body composition in relation to 14 cardiovascular conditions in UK Biobank: A Mendelian randomization study

| **Table S1** | Definitions and sources of information for outcomes in UK Biobank | Page 2 |
| --- | --- | --- |
| **Table S2** | Single-nucleotide polymorphisms used as instrumental variables in the Mendelian randomization analyses of body mass index | Page 3 |
| **Table S2** | Single-nucleotide polymorphisms used as instrumental variables in the multivariable Mendelian randomization analyses of fat mass and fat-free mass indices | Page 6 |
| **Table S4** | Statistical power in the Mendelian randomization analyses of body mass index in relation to different outcomes | Page 8 |
| **Table S5** | Associations between genetically predicted 1 kg/m^2^ increase in body mass index and cardiovascular diseases in sensitivity analyses using the weighted median and MR-Egger methods | Page 9 |
|  |  |  |

**Table S1.** Definitions and sources of information for outcomes in UK Biobank

|  | **Number**  **of cases** | **ICD-9 diagnosis** | **ICD-10 diagnosis** | **OPCS procedure** | **Self-report^a^** |
| --- | --- | --- | --- | --- | --- |
| **Cerebrovascular diseases** |  |  |  |  |  |
| Ischaemic stroke | 3554 | 434.X, 436.X | I63.X, I64.X |  | 20002 |
| Transient ischemic attack | 3485 | 435.X | G45.X |  | 20002 |
| Intracerebral hemorrhage | 1655 | 431.X | I61.X |  | 20002 |
| Subarachnoid hemorrhage | 1834 | 430.X | I60.X |  | 20002 |
| **Aortic aneurysms** |  |  |  |  |  |
| Abdominal aortic aneurysm | 758 | 441.3, 441.4 | I71.3, I71.4 | L19.4, L19.5 | 20002 |
| Thoracic aortic aneurysm | 231 | 441.1, 441.2 | I71.1, I71.2 |  | 20002 |
| **Thrombotic diseases** |  |  |  |  |  |
| Deep vein thrombosis | 8891 | 451.1 | I80.2 | L90.2 | 20002, 6152 |
| Pulmonary embolism | 5097 | 415.1 | I26.X |  | 20002, 6152 |
| **Other CVDs** |  |  |  |  |  |
| Coronary artery disease | 24 531 | 410.X, 411.X, 412.X, 414.0, 414.8, 414.9 | I21.X, I22.X, I23.X, I24.X, I25.1, I25.2, I25.5, I25.6, I25.8, I25.9 | K40.X, K41.X, K42.X, K43.X, K44.X, K45.X, K46.X, K49.X, K50.1, K50.2, K50.4, K75.X | 20002, 20004, 6150 |
| Aortic valve stenosis | 1252 |  | I35.0, I35.2 |  | 20002 |
| Atrial fibrillation | 13 538 | 427.3 | I48 |  | 20002 |
| Heart failure | 4803 | 402.01, 402.11, 402.91, 404.01, 404.11, 404.91, 404.03, 404.13, 404.93, 428.X | I11.0, I13.0, I13.2, I50.X |  | 20002 |
| Peripheral vascular disease | 3514 | 443.8, 443.9 | I73.8, I73.9 |  | 20002 |
| Arterial hypertension | 119 500 | 401.X | I10 |  | 20002, 6150, 6177 |

CVDs, cardiovascular diseases; ICD International Classification of Disease; OPCS, Office of Population Censuses and Surveys Classification of Surgical Operations and Procedures.

^a^Numbers refer to data codes used in UK Biobank: 6150/6152 = Health condition diagnosed by doctor (self-reported from touchscreen); 6177 = Medication for health condition (self-reported from touchscreen); 20002 = Non-cancer illness code (self-reported from interview with nurse); 20004 = Surgical operation code (self-reported from interview with nurse).

**Table S2.** Single-nucleotide polymorphisms used as instrumental variables in the Mendelian randomization analyses of body mass index

| **SNP** | **Chr** | **EA** | **NEA** | **Beta^a^** | **SE** | **Nearby gene** |
| --- | --- | --- | --- | --- | --- | --- |
| rs543874 | 1 | G | A | 0.0482 | 0.0039 | *SEC16B* |
| rs3101336 | 1 | C | T | 0.0334 | 0.0031 | *NEGR1* |
| rs12566985 | 1 | G | A | 0.0242 | 0.0031 | *FPGT-TNNI3K* |
| rs17024393 | 1 | C | T | 0.0658 | 0.0088 | *GNAT2* |
| rs657452 | 1 | A | G | 0.0227 | 0.0031 | *AGBL4* |
| rs11165643 | 1 | T | C | 0.0218 | 0.0031 | *PTBP2* |
| rs12401738 | 1 | A | G | 0.0211 | 0.0033 | *FUBP1* |
| rs2820292 | 1 | C | A | 0.0195 | 0.0031 | *NAV1* |
| rs11583200 | 1 | C | T | 0.0177 | 0.0031 | *ELAVL4* |
| rs977747 | 1 | T | G | 0.0167 | 0.0031 | *TAL1* |
| rs13021737 | 2 | G | A | 0.0601 | 0.0040 | *TMEM18* |
| rs10182181 | 2 | G | A | 0.0307 | 0.0031 | *ADCY3* |
| rs1016287 | 2 | T | C | 0.0229 | 0.0034 | *LINC01122* |
| rs7599312 | 2 | G | A | 0.0220 | 0.0034 | *ERBB4* |
| rs11126666 | 2 | A | G | 0.0207 | 0.0034 | *KCNK3* |
| rs492400 | 2 | C | T | 0.0158 | 0.0031 | *USP37* |
| rs2176040 | 2 | A | G | 0.0141 | 0.0031 | *LOC646736* |
| rs1528435 | 2 | T | C | 0.0178 | 0.0031 | *UBE2E3* |
| rs11688816 | 2 | G | A | 0.0172 | 0.0031 | *EHBP1* |
| rs2121279 | 2 | T | C | 0.0245 | 0.0044 | *LRP1B* |
| rs17203016 | 2 | G | A | 0.0210 | 0.0039 | *CREB1* |
| rs1460676 | 2 | C | T | 0.0197 | 0.0040 | *FIGN* |
| rs1516725 | 3 | C | T | 0.0451 | 0.0046 | *ETV5* |
| rs13078960 | 3 | G | T | 0.0297 | 0.0039 | *CADM2* |
| rs2365389 | 3 | C | T | 0.0200 | 0.0031 | *FHIT* |
| rs16851483 | 3 | T | G | 0.0483 | 0.0077 | *RASA2* |
| rs6804842 | 3 | G | A | 0.0185 | 0.0031 | *RARB* |
| rs3849570 | 3 | A | C | 0.0188 | 0.0034 | *GBE1* |
| rs10938397 | 4 | G | A | 0.0402 | 0.0031 | *GNPDA2* |
| rs13107325 | 4 | T | C | 0.0477 | 0.0068 | *SLC39A8* |
| rs17001654 | 4 | G | C | 0.0306 | 0.0053 | *SCARB2* |
| rs11727676 | 4 | T | C | 0.0358 | 0.0064 | *HHIP* |
| rs2112347 | 5 | T | G | 0.0261 | 0.0031 | *POC5* |
| rs7715256 | 5 | G | T | 0.0163 | 0.0031 | *GALNT10* |
| rs2207139 | 6 | G | A | 0.0447 | 0.0040 | *TFAP2B* |
| rs205262 | 6 | G | A | 0.0221 | 0.0035 | *C6orf106* |
| rs13191362 | 6 | A | G | 0.0277 | 0.0048 | *PARK2* |
| rs2033529 | 6 | G | A | 0.0190 | 0.0033 | *TDRG1* |
| rs9400239 | 6 | C | T | 0.0188 | 0.0033 | *FOXO3* |
| rs9374842 | 6 | T | C | 0.0187 | 0.0035 | *LOC285762* |
| rs13201877 | 6 | G | A | 0.0233 | 0.0045 | *IFNGR1* |
| rs9641123 | 7 | C | G | 0.0191 | 0.0038 | *CALCR* |
| rs1167827 | 7 | G | A | 0.0202 | 0.0033 | *HIP1* |
| rs2245368 | 7 | C | T | 0.0317 | 0.0057 | *PMS2L11* |
| rs6465468 | 7 | T | G | 0.0166 | 0.0035 | *ASB4* |
| rs17405819 | 8 | T | C | 0.0224 | 0.0033 | *HNF4G* |
| rs16907751 | 8 | C | T | 0.0350 | 0.0066 | *ZBTB10* |
| rs2033732 | 8 | C | T | 0.0192 | 0.0035 | *RALYL* |
| rs10968576 | 9 | G | A | 0.0249 | 0.0033 | *LINGO2* |
| rs1928295 | 9 | T | C | 0.0188 | 0.0031 | *TLR4* |
| rs4740619 | 9 | T | C | 0.0179 | 0.0031 | *C9orf93* |
| rs10733682 | 9 | A | G | 0.0174 | 0.0031 | *LMX1B* |
| rs6477694 | 9 | C | T | 0.0174 | 0.0031 | *EPB41L4B* |
| rs7903146 | 10 | C | T | 0.0234 | 0.0034 | *TCF7L2* |
| rs17094222 | 10 | C | T | 0.0249 | 0.0038 | *HIF1AN* |
| rs11191560 | 10 | C | T | 0.0308 | 0.0053 | *NT5C2* |
| rs7899106 | 10 | G | A | 0.0395 | 0.0071 | *GRID1* |
| rs11030104 | 11 | A | G | 0.0414 | 0.0038 | *BDNF* |
| rs3817334 | 11 | T | C | 0.0262 | 0.0031 | *MTCH2* |
| rs12286929 | 11 | G | A | 0.0217 | 0.0031 | *CADM1* |
| rs4256980 | 11 | G | C | 0.0209 | 0.0031 | *TRIM66* |
| rs2176598 | 11 | T | C | 0.0198 | 0.0036 | *HSD17B12* |
| rs7138803 | 12 | A | G | 0.0315 | 0.0031 | *BCDIN3D* |
| rs11057405 | 12 | G | A | 0.0307 | 0.0055 | *CLIP1* |
| rs12429545 | 13 | A | G | 0.0334 | 0.0047 | *OLFM4* |
| rs1441264 | 13 | A | G | 0.0175 | 0.0032 | *MIR548A2* |
| rs9540493 | 13 | A | G | 0.0172 | 0.0033 | *MIR548X2* |
| rs7141420 | 14 | T | C | 0.0235 | 0.0031 | *NRXN3* |
| rs10132280 | 14 | C | A | 0.0230 | 0.0034 | *STXBP6* |
| rs12885454 | 14 | C | A | 0.0207 | 0.0033 | *PRKD1* |
| rs11847697 | 14 | T | C | 0.0492 | 0.0084 | *PRKD1* |
| rs16951275 | 15 | T | C | 0.0311 | 0.0037 | *MAP2K5* |
| rs7164727 | 15 | T | C | 0.0180 | 0.0033 | *LOC100287559* |
| rs3736485 | 15 | A | G | 0.0176 | 0.0031 | *DMXL2* |
| rs1558902 | 16 | A | T | 0.0818 | 0.0031 | *FTO* |
| rs3888190 | 16 | A | C | 0.0309 | 0.0031 | *ATP2A1* |
| rs12446632 | 16 | G | A | 0.0403 | 0.0046 | *GPRC5B* |
| rs758747 | 16 | T | C | 0.0225 | 0.0037 | *NLRC3* |
| rs9925964 | 16 | A | G | 0.0192 | 0.0031 | *KAT8* |
| rs2650492 | 16 | A | G | 0.0207 | 0.0035 | *SBK1* |
| rs2080454 | 16 | C | A | 0.0168 | 0.0031 | *CBLN1* |
| rs4787491 | 16 | G | A | 0.0159 | 0.0034 | *INO80E* |
| rs12940622 | 17 | G | A | 0.0182 | 0.0031 | *RPTOR* |
| rs1000940 | 17 | G | A | 0.0192 | 0.0034 | *RABEP1* |
| rs9914578 | 17 | G | C | 0.0201 | 0.0038 | *SMG6* |
| rs6567160 | 18 | C | T | 0.0556 | 0.0036 | *MC4R* |
| rs7239883 | 18 | G | A | 0.0164 | 0.0031 | *LOC284260* |
| rs7243357 | 18 | T | G | 0.0217 | 0.0040 | *GRP* |
| rs1808579 | 18 | C | T | 0.0167 | 0.0031 | *C18orf8* |
| rs2287019 | 19 | C | T | 0.0360 | 0.0042 | *QPCTL* |
| rs3810291 | 19 | A | G | 0.0283 | 0.0036 | *ZC3H4* |
| rs2075650 | 19 | A | G | 0.0258 | 0.0045 | *TOMM40* |
| rs29941 | 19 | G | A | 0.0182 | 0.0033 | *KCTD15* |
| rs17724992 | 19 | A | G | 0.0194 | 0.0035 | *PGPEP1* |
| rs6091540 | 20 | C | T | 0.0188 | 0.0035 | *ZFP64* |
| rs2836754 | 21 | C | T | 0.0164 | 0.0032 | *ETS2* |

Chr, chromosome; EA, effect allele; NEA, non-effect allele; SE, standard error; SNP, single-nucleotide polymorphisms.

^a^Standard deviation change in BMI per additional effect allele.

**Table S3.** Single-nucleotide polymorphisms used as instrumental variables in the multivariable Mendelian randomization analyses of fat mass and fat-free mass indices

|  |  | **Fat mass index** | |  | **Fat-free mass index** | |
| --- | --- | --- | --- | --- | --- | --- |
| **SNP** | **EA** | **Beta** | **SE** |  | **Beta** | **SE** |
| rs4846204 | T | 0.00568763 | 0.00353654 |  | -0.0132865 | 0.00353955 |
| rs2273368 | T | -0.00950088 | 0.00296335 |  | 0.00467889 | 0.00296596 |
| rs11205303 | C | 0.011527 | 0.00239791 |  | -0.0187882 | 0.00239987 |
| rs4971091 | T | -0.00277839 | 0.00242915 |  | -0.0128365 | 0.00243117 |
| rs180921974 | G | -0.00569087 | 0.00796463 |  | 0.048922 | 0.00797114 |
| rs545608 | C | 0.0426564 | 0.00291473 |  | 0.033517 | 0.0029176 |
| rs991967 | C | 0.00397586 | 0.00260548 |  | -0.0009915 | 0.00260775 |
| rs2820443 | C | 0.0199628 | 0.00257539 |  | -0.0087129 | 0.0025778 |
| rs10916174 | A | 0.000977569 | 0.00340923 |  | 0.0100294 | 0.00341215 |
| rs62107261 | C | -0.0724923 | 0.00552507 |  | -0.071529 | 0.0055299 |
| rs6731872 | G | 0.0414591 | 0.00311263 |  | 0.0405763 | 0.00311536 |
| rs754537 | T | -0.027853 | 0.00235721 |  | -0.0186913 | 0.00235951 |
| rs17511102 | T | -0.00683403 | 0.00413242 |  | -0.0146531 | 0.00413596 |
| rs7562173 | C | -0.0134856 | 0.00242180 |  | -0.00854795 | 0.00242396 |
| rs3791679 | G | 0.000215618 | 0.00281051 |  | 0.0148106 | 0.00281284 |
| rs13011472 | G | -0.00645166 | 0.00236683 |  | -0.0126982 | 0.00236881 |
| rs4521268 | T | 0.000388904 | 0.00263508 |  | -0.00570584 | 0.00263735 |
| rs1986599 | G | 0.0209351 | 0.00375217 |  | 0.0257344 | 0.00375535 |
| rs9853018 | T | 0.00633379 | 0.00236909 |  | -0.00417625 | 0.00237116 |
| rs2241069 | C | 0.00665045 | 0.00236920 |  | 0.00718266 | 0.00237125 |
| rs79334166 | G | 0.0152242 | 0.00349048 |  | 0.00657099 | 0.00349359 |
| rs4694504 | G | 0.00424849 | 0.00236034 |  | -0.0169092 | 0.00236223 |
| rs994014 | C | 0.00407693 | 0.00255666 |  | -0.00706258 | 0.00255886 |
| rs7680661 | A | -0.000909614 | 0.00314511 |  | -0.0108018 | 0.00314779 |
| rs465983 | G | 0.00318318 | 0.00276783 |  | -0.00796722 | 0.0027702 |
| rs34341 | T | 0.0220796 | 0.00238313 |  | 0.0161727 | 0.00238533 |
| rs115912456 | G | -0.0190078 | 0.00591417 |  | 0.0290739 | 0.00591919 |
| rs1317415 | C | 0.00890126 | 0.00256289 |  | -0.0032274 | 0.00256515 |
| rs351855 | A | 0.00100048 | 0.00257940 |  | 0.0146507 | 0.00258153 |
| rs888762 | A | -0.000453192 | 0.00251587 |  | 0.00794187 | 0.00251802 |
| rs41271299 | T | 0.0000717 | 0.00531736 |  | -0.0118184 | 0.00532195 |
| rs9358913 | G | -0.0195693 | 0.00267934 |  | 0.00310442 | 0.00268186 |
| rs9469762 | A | 0.0243286 | 0.00713169 |  | 0.0133184 | 0.00713797 |
| rs2492863 | A | 0.0292846 | 0.00339097 |  | 0.0150526 | 0.00339417 |
| rs2635727 | C | 0.0242421 | 0.00277930 |  | 0.0151325 | 0.00278189 |
| rs314263 | T | -0.000787961 | 0.00252178 |  | 0.0169313 | 0.00252381 |
| rs6570507 | A | 0.00421113 | 0.00260722 |  | 0.0103901 | 0.00260944 |
| rs2982708 | C | -0.000416001 | 0.00263727 |  | -0.00274577 | 0.00263956 |
| rs798491 | G | -0.008832 | 0.00257166 |  | 0.00884354 | 0.00257389 |
| rs3823974 | C | -0.000387929 | 0.00240226 |  | 0.00641643 | 0.00240432 |
| rs481806 | T | -0.00382811 | 0.00258527 |  | 0.0112596 | 0.00258746 |
| rs56282717 | A | -0.00906299 | 0.00275538 |  | -0.0154138 | 0.0027577 |
| rs2044387 | A | 0.0120543 | 0.00239675 |  | 0.0157855 | 0.00239877 |
| rs12546366 | C | -0.0133157 | 0.0023716 |  | -0.0152213 | 0.00237363 |
| rs4733727 | T | 0.00378111 | 0.00236202 |  | 0.00745621 | 0.00236405 |
| rs10962638 | A | -0.00518625 | 0.00329827 |  | 0.00676688 | 0.00330113 |
| rs35344761 | A | -0.00583235 | 0.00362286 |  | 0.0160285 | 0.00362592 |
| rs7039458 | A | -0.00429684 | 0.00272317 |  | 0.0107874 | 0.00272549 |
| rs3780327 | G | -0.00604955 | 0.002847 |  | -0.0001631 | 0.00284949 |
| rs1138714 | G | 0.00941741 | 0.00239498 |  | 0.0117718 | 0.00239704 |
| rs10769282 | G | -0.0217916 | 0.00256266 |  | -0.00754611 | 0.00256512 |
| rs71455793 | A | -0.0126712 | 0.00563878 |  | -0.00500981 | 0.00564371 |
| rs4980661 | A | -0.00768201 | 0.00235715 |  | -0.0147272 | 0.00235911 |
| rs1789166 | A | 0.0079086 | 0.00246377 |  | 0.00964178 | 0.0024659 |
| rs34716573 | C | 0.00245826 | 0.00247382 |  | -0.00539166 | 0.00247596 |
| rs11049566 | T | 0.0021472 | 0.0026036 |  | 0.00629556 | 0.00260584 |
| rs7306275 | A | 0.0235115 | 0.00244152 |  | 0.0152495 | 0.00244382 |
| rs11614785 | G | 0.00227946 | 0.00250889 |  | -0.0114692 | 0.00251101 |
| rs6489111 | G | 0.0127331 | 0.00246935 |  | 0.0066576 | 0.00247156 |
| rs6492538 | C | -0.00434959 | 0.0028499 |  | 0.0046882 | 0.00285237 |
| rs71420186 | A | 0.0111809 | 0.00474192 |  | -0.0135572 | 0.00474603 |
| rs35874463 | G | 0.00131541 | 0.00504661 |  | -0.0139676 | 0.00505094 |
| rs12905253 | A | 0.000833859 | 0.00235669 |  | 0.0165561 | 0.00235857 |
| rs11856122 | A | 0.01141 | 0.00235806 |  | -0.0255185 | 0.0023598 |
| rs28584580 | G | 0.00921397 | 0.00689559 |  | 0.0349408 | 0.00690136 |
| rs3817428 | G | -0.00605682 | 0.00266925 |  | 0.0227523 | 0.00267132 |
| rs72755233 | A | -0.00209186 | 0.00373354 |  | 0.0319296 | 0.00373641 |
| rs4988781 | C | 0.000982696 | 0.00251727 |  | 0.0105241 | 0.00251939 |
| rs8050894 | G | -0.0262074 | 0.00242399 |  | -0.0109009 | 0.00242642 |
| rs8050894 | T | -0.030769 | 0.14659 |  | 0.0111797 | 0.146717 |
| rs55872725 | T | 0.0565272 | 0.00239922 |  | 0.0593398 | 0.00240112 |
| rs8057620 | T | 0.0189601 | 0.00236844 |  | 0.00643719 | 0.00237069 |
| rs2071167 | T | 0.0183232 | 0.00278498 |  | -0.00549673 | 0.00278755 |
| rs2074188 | G | 0.00381863 | 0.00236372 |  | -0.00566998 | 0.00236577 |
| rs28394864 | A | 0.0025285 | 0.00237384 |  | 0.0128869 | 0.0023758 |
| rs4800148 | A | 0.00122248 | 0.00284806 |  | -0.00100824 | 0.00285054 |
| rs7236575 | A | 0.0105525 | 0.00342257 |  | 0.00362767 | 0.00342558 |
| rs62621197 | T | -0.00638871 | 0.00648015 |  | 0.0292546 | 0.0064856 |
| rs10402308 | A | 0.00731973 | 0.00307487 |  | 0.00733223 | 0.00307755 |
| rs6085551 | C | -0.00416452 | 0.0023635 |  | -0.00662193 | 0.00236554 |
| rs2145270 | T | 0.0122696 | 0.00242437 |  | 0.0187586 | 0.00242636 |
| rs143384 | G | -0.00490453 | 0.00240128 |  | 0.000873025 | 0.00240338 |

EA, effect allele; SE, standard error; SNP, single-nucleotide polymorphisms.

**Table S4.** Statistical power in the Mendelian randomization analyses of body mass index in relation to different outcomes per standard deviation (about 4.8 kg/m^2^) increase in body mass index

|  |  | **Power at different odds ratios and α=0.05** | | | |
| --- | --- | --- | --- | --- | --- |
| **Outcome** | **Cases^a^** | **1.2** | **1.5** | **1.8** | **2.0** |
| **Cerebrovascular diseases** |  |  |  |  |  |
| Ischemic stroke | 3554 | 0.51 | 1.00 | 1.00 | 1.00 |
| Transient ischemic attack | 3485 | 0.51 | 1.00 | 1.00 | 1.00 |
| Intracerebral hemorrhage | 1655 | 0.29 | 0.94 | 1.00 | 1.00 |
| Subarachnoid hemorrhage | 1834 | 0.29 | 0.94 | 1.00 | 1.00 |
| **Aortic aneurysms** |  |  |  |  |  |
| Abdominal aortic aneurysm | 758 | 0.14 | 0.60 | 0.94 | 1.00 |
| Thoracic aortic aneurysm | 231 | 0.08 | 0.23 | 0.50 | 0.68 |
| **Thrombotic diseases** |  |  |  |  |  |
| Deep vein thrombosis | 8891 | 0.86 | 1.00 | 1.00 | 1.00 |
| Pulmonary embolism | 5097 | 0.65 | 1.00 | 1.00 | 1.00 |
| **Other outcomes** |  |  |  |  |  |
| Coronary artery disease | 24 531 | 1.00 | 1.00 | 1.00 | 1.00 |
| Aortic valve stenosis | 1252 | 0.19 | 0.78 | 0.99 | 1.00 |
| Atrial fibrillation | 13 538 | 1.00 | 1.00 | 1.00 | 1.00 |
| Heart failure | 4803 | 1.00 | 1.00 | 1.00 | 1.00 |
| Peripheral artery disease | 3514 | 0.51 | 1.00 | 1.00 | 1.00 |
| Arterial hypertension | 119 500 | 1.00 | 1.00 | 1.00 | 1.00 |

BMI, body mass index.

^a^Total number of participants is 367 703.

**Table S5.** Associations between genetically predicted 1 kg/m^2^ increase in body mass index and cardiovascular diseases in sensitivity analyses using the weighted median and MR-Egger methods

|  | **Weighted median** | |  | **MR-Egger** | | | |  |
| --- | --- | --- | --- | --- | --- | --- | --- | --- |
| **Outcome** | **OR (95% CI)** | ***P* value** |  | **OR (95% CI)** | ***P* value** |  | **Intercept^a^** | ***P* value** |
| **Cerebrovascular diseases** |  |  |  |  |  |  |  |  |
| Ischemic stroke | 1.02 (0.95-1.10) | 0.53 |  | 0.99 (0.89-1.09) | 0.80 |  | 0.006 (-0.007 to 0.019) | 0.39 |
| Transient ischemic attack | 1.03 (0.97-1.10) | 0.34 |  | 1.13 (1.01-1.26) | 0.04 |  | -0.014 (-0.029 to 0.000) | 0.05 |
| Intracerebral hemorrhage | 1.03 (0.93-1.15) | 0.56 |  | 0.99 (0.85-1.16) | 0.92 |  | 0.005 (-0.015 to 0.025) | 0.61 |
| Subarachnoid hemorrhage | 1.03 (0.94-1.13) | 0.55 |  | 1.01 (0.87-1.17) | 0.92 |  | 0.007 (-0.011 to 0.026) | 0.77 |
| **Aortic aneurysms** |  |  |  |  |  |  |  |  |
| Abdominal aortic aneurysm | 0.96 (0.83-1.11) | 0.57 |  | 0.99 (0.78-1.24) | 0.90 |  | 0.010 (-0.020 to 0.040) | 0.51 |
| Thoracic aortic aneurysm | 0.97 (0.73-1.29) | 0.84 |  | 0.96 (0.64-1.44) | 0.83 |  | 0.001 (-0.051 to 0.054) | 0.96 |
| **Thrombotic diseases** |  |  |  |  |  |  |  |  |
| Deep vein thrombosis | 1.09 (1.04-1.14) | 0.001 |  | 1.13 (1.05-1.21) | 0.001 |  | -0.004 (-0.012 to 0.005) | 0.42 |
| Pulmonary embolism | 1.05 (0.99-1.12) | 0.11 |  | 1.04 (0.94-1.14) | 0.46 |  | 0.003 (-0.009 to 0.016) | 0.59 |
| **Other CVDs** |  |  |  |  |  |  |  |  |
| Coronary artery disease | 1.04 (1.01-1.07) | 0.003 |  | 0.99 (0.93-1.06) | 0.87 |  | 0.010 (0.001 to 0.019) | 0.03 |
| Aortic valve stenosis | 1.17 (1.04-1.32) | 0.01 |  | 1.12 (0.94-1.33) | 0.21 |  | 0.001 (-0.021 to 0.023) | 0.94 |
| Atrial fibrillation | 1.04 (1.01-1.08) | 0.03 |  | 0.99 (0.93-1.04) | 0.63 |  | 0.010 (0.003 to 0.018) | 0.004 |
| Heart failure | 1.10 (1.03-1.16) | 0.002 |  | 1.05 (0.95-1.16) | 0.34 |  | 0.008 (-0.005 to 0.022) | 0.21 |
| Peripheral artery disease | 1.10 (1.02-1.18) | 0.01 |  | 1.08 (0.97-1.20) | 0.16 |  | -0.001 (-0.014 to 0.013) | 0.92 |
| Arterial hypertension | 1.11 (1.09-1.13) | 3.9×10^-30^ |  | 1.06 (1.01-1.12) | 0.02 |  | 0.004 (-0.003 to 0.011) | 0.23 |

CI, confidence interval; CVDs, cardiovascular diseases; OR odds ratio.

^a^The MR-Egger intercept quantifies the effect of directional pleiotropy. Values that significantly differ from zero provide evidence that the BMI-associated single-nucleotide polymorphisms may influence the outcome through other pathways than through BMI.
